# Accuracy, Validity, and Reliability of an Electronic Visual Analog Scale for Pain on a Touch Screen Tablet in Healthy Older Adults: A Clinical Trial

**DOI:** 10.2196/ijmr.4910

**Published:** 2016-01-14

**Authors:** Marie-Louise Bird, Michele L Callisaya, John Cannell, Timothy Gibbons, Stuart T Smith, Kiran DK Ahuja

**Affiliations:** ^1^ School of Health Sciences University of Tasmania Launceston Australia; ^2^ Menzies Institute of Medical Research University of Tasmania Hobart Australia; ^3^ Department of Health and Human Services Launceston Australia; ^4^ University of the Sunshine Coast Sunshine Coast Australia

**Keywords:** pain, VAS, technology, scale

## Abstract

**Background:**

New technology for clinical data collection is rapidly evolving and may be useful for both researchers and clinicians; however, this new technology has not been tested for accuracy, reliability, or validity.

**Objective:**

This study aims to test the accuracy of visual analog scale (VAS) for pain on a newly designed application on the iPad (iPadVAS) and measure the reliability and validity of iPadVAS compared to a paper copy (paperVAS).

**Methods:**

Accuracy was determined by physically measuring an iPad scale on screen and comparing it to the results from the program, with a researcher collecting 101 data points. A total of 22 healthy community dwelling older adults were then recruited to test reliability and validity. Each participant completed 8 VAS (4 using each tool) in a randomized order. Reliability was measured using interclass correlation coefficient (ICC) and validity measured using Bland-Altman graphs and correlations.

**Results:**

Of the measurements for accuracy, 64 results were identical, 2 results were manually measured as being 1 mm higher than the program, and 35 as 1 mm lower. Reliability for the iPadVAS was excellent with individual ICC 0.90 (95% CI 0.82-0.95) and averaged ICC 0.97 (95% CI 0.95-1.0) observed. Linear regression demonstrated a strong relationship with a small negative bias towards the iPad (−2.6, SD 5.0) with limits of agreement from −12.4 to 7.1.

**Conclusions:**

The iPadVAS provides a convenient, user-friendly, and efficient way of collecting data from participants in measuring their current pain levels. It has potential use in documentation management and may encourage participatory healthcare.

**Trial Registration:**

Australia New Zealand Clinical Trials Registry (ANZCTR): 367297; https://www.anzctr.org.au/Trial/Registration/TrialReview.aspx?id=367297&isReview=true (Archived by Webcite at http://www.webcitation.org/6d9xYoUbD).

## Introduction

The health care sector is poised at the cusp of a transformation from being reactive to disease and injury toward proactive prevention, where the ultimate goal is to maximize individual health rather than treat disease. Ready access to medical information combined with ubiquitous sensing, quantified self, mobile computing, and social networking technologies empowers individuals to participate in their own health and well-being. According to Hood and colleagues [1], active participation by individuals is a central component of the revolution in health care and wellness.

The ability to measure pain objectively forms an important part of health care, both in chronic health monitoring and in acute settings, to determine changes in patient clinical presentation and the effectiveness of interventions aimed at alleviating pain. Visual analog scales (VAS) for collecting pain data in the traditional paper-based format have been shown to be accurate, valid, reliable, and reproducible [2] across a range of settings. Using paper-based versions of VAS scales requires application of the scale in a standard manner, measurement of the value with a ruler, and then copying of the value into notes or electronic databases. This manual entry is time-consuming and has the potential for transcription or typing errors. When the researcher or clinician has to travel, paper versions of data collection are bulky and can be problematic for ensuring secure storage during transport.

Collecting the pain data electronically streamlines data measurement and management. Previously, electronic data collection using hand-held devices (eg, personal digital assistants or laptop computers) for VAS for pain has found values to be equivalent to paper-based tools; however, these electronic tools were costly [3] and differed from the paper version in the method of interacting with screen and sensations measured [4].

Costs for new technology including hand-held tablets have decreased in recent years. These devices have the benefit of a user-friendly touch screen interface. With appropriate applications, data collected on a touch screen can be automatically measured and exported to a database for secure storage within the device and can easily be emailed to the researcher or clinician when access to the Internet is available. However, this new technology has not been tested for accuracy and reliability or compared to the paper-based gold standard for validity.

We assessed the accuracy of VAS for pain on an iPad (iPadVAS), measured and compared the reliability of iPadVAS to a paper copy (paperVAS), and validated the iPadVAS against paperVAS in a healthy community group.

## Methods

### Accuracy Study

A single researcher drew a line across the iPadVAS line with 1 finger 100 times using all parts of the scale. After each effort, the value was measured on the 100 mm line with a ruler that had 1 mm gradations marked on it; the number corresponding to the value of the mark (ie, a number between 0 and 100) was recorded in an Excel spreadsheet (Microsoft Corp). The researcher was blinded to the results generated by the application at the time. Measures by the researcher were then compared to the data produced by the algorithm in the iPad application.

### Tools

#### Description of paperVAS

The paperVAS was administered mounted on a clipboard and completed using a pen (0.7 mm tip width) on a line 100 mm in length and 0.75 mm high with no markings on the scale except *No pain* on the left and *Worst possible pain* on the right [2]. To preserve the dimensions of the lines, paper copies were printed and not photocopied.

#### Description of iPadVAS

**Figure 1:**
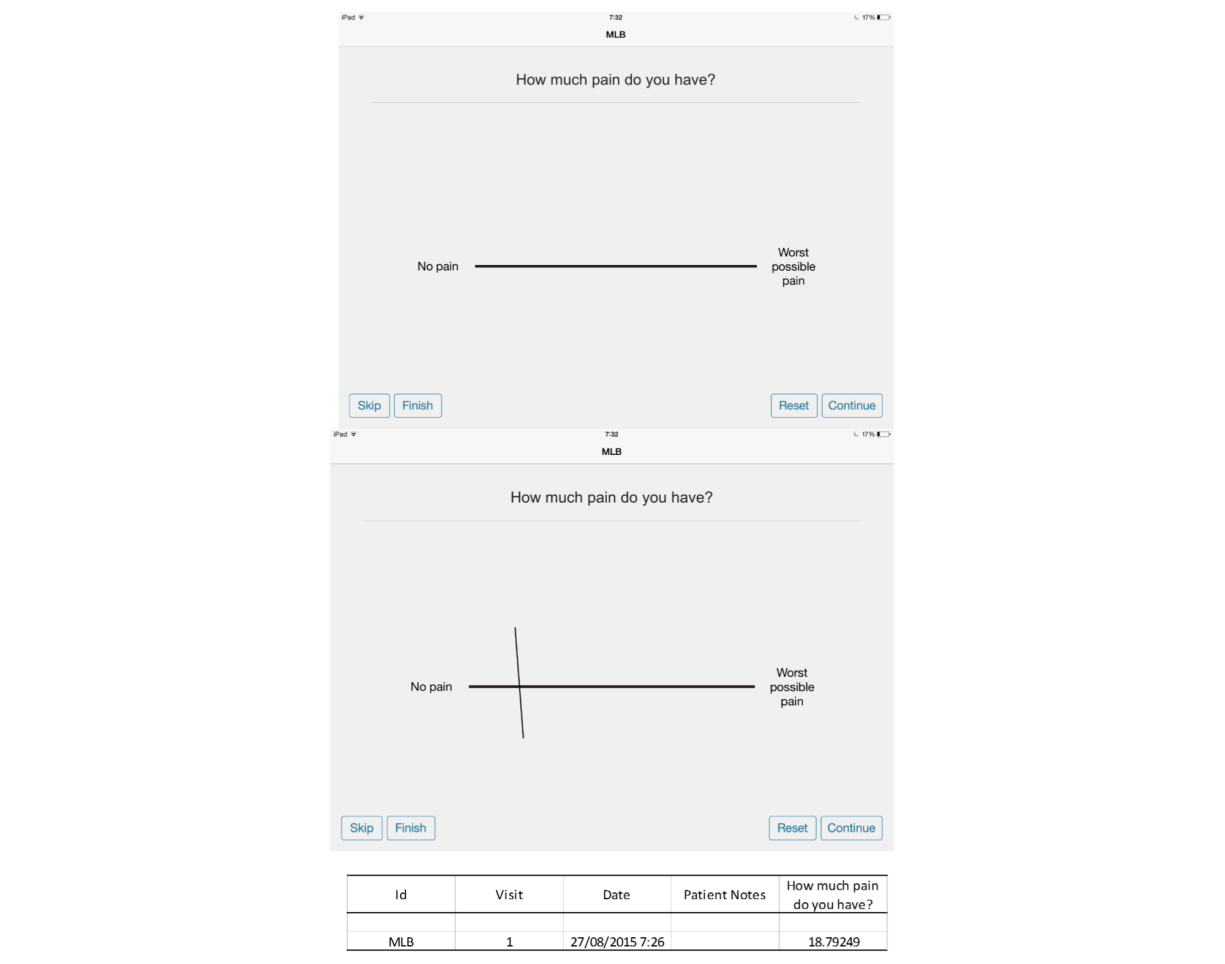
Screen capture of a blank iPadVAS, a completed iPadVAS, and the CSV data output file.

iPadVAS was administered on an iPad 2 and completed by the participant using their finger on the screen using an application developed by the research team [5]. Similar to paperVAS, the iPadVAS was 100.06 mm long and 0.96 mm high with no markings on the scale except *No pain* on the left and *Worst possible pain* on the right end of the scale. The line that the fingertip generated on the screen was 0.38 mm wide. To preserve the dimensions of the lines, the application was locked in landscape orientation and could not be used in portrait orientation. User interface elements could not be scaled or rotated. Figure 1 shows a screen capture of a blank iPadVAS and an example of a completed iPadVAS with the data output obtained as a CSV file.

### Comparative Study

#### Participants and Setting

This was a single center study conducted in healthy older adults (ages 65-85 years) at the Exercise Physiology Clinic at the University of Tasmania, Launceston, Australia. Participants who were enrolled for group Pilates classes were invited to take part in the study. The exclusion criterion was people who self-reported inadequate vision to complete the tasks. This study was approved by Human Medical Research Ethics Committee (Tasmania) Network (H0014062). The study is registered with ANZCTR (367297). Written informed consent was obtained from each participant (see Multimedia Appendix 1 for the CONSORT checklist).

#### Procedure

The study involved two tasks: completion of a paper-based and an electronic VAS for pain. Tests were administered in a randomized order in a single session lasting 30 minutes. Data were collected between November 2014 and April 2015. Each participant chose a token with eyes closed and the color of token selected determined the order (blue: iPadVAS first; red: paperVAS first) of the tasks. Each study participant was given instruction to draw a line through the line on the paper or iPad that corresponded to their current level of pain. They were provided with a demonstration of both tools. Each participant completed both tasks four times. After each effort, the results for the task on iPadVAS were saved and the screen reset so that the previous data were not available for comparison to the participant. Similarly, for the paperVAS, information from previous efforts was not available to the participants.

#### Sample Size

A change of 13 points in VAS for pain is considered as a clinically significant change [6]. A priori sample size calculation indicated that a sample size of 21 would provide a power of 90% (alpha .05; SD 18) to detect a mean difference of 13 between iPadVAS and paperVAS.

#### Statistical Analyses

All analyses were performed using Stata Intercooled software version 13 (StataCorp LP). The accuracy of the application algorithm to determine the value on the scale was analyzed by comparing data manually measured using a paired *t test* to determine any differences (*P*=.05). Linear regression was used to determine the relationship between these two methods of data collection.

Reliability was measured for both the iPadVAS and paperVAS using absolute agreement interclass correlation coefficient (ICC, 95% CI), and linear regression was used to determine the relationship between these two methods of data collection. Reliability was reported as excellent (ICC 0.90 and higher), good (ICC between 0.80 and 0.89), moderate (ICC between 0.70 and 0.79), or low (ICC less than 0.70) [7]. Validity of the data recorded using the iPadVAS was compared to the paperVAS using Bland-Altman graphs (measuring bias and limits of agreement) and correlations to describe the relationship.

## Results

### Accuracy Study

A researcher compared 101 data points by examining the difference between manual measurement and the calculated measurement produced by the iPad application program. Accuracy was high with 64 identical results, 2 manually measured results 1 mm higher, and 35 lower by 1 mm than the iPad program. Student *t test* indicated a nonsignificant difference of 0.4 mm (*P*=.35).

**Figure 2:**
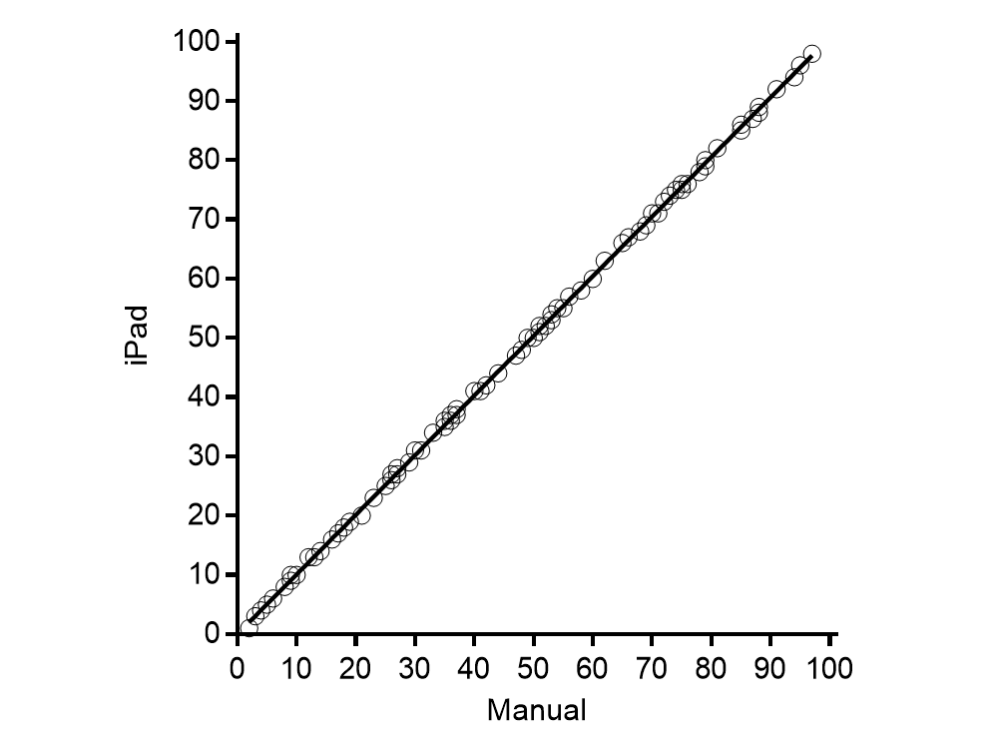
Correlation of VAS scores between manual and iPad application program.

Linear regression showed high correlation of the scores between the two measurement techniques (*R* ^2^=.9998) equation Y=1.007 × X + 0.02285 (Figure 2).

### Comparative Study

#### Reliability

**Figure 3:**
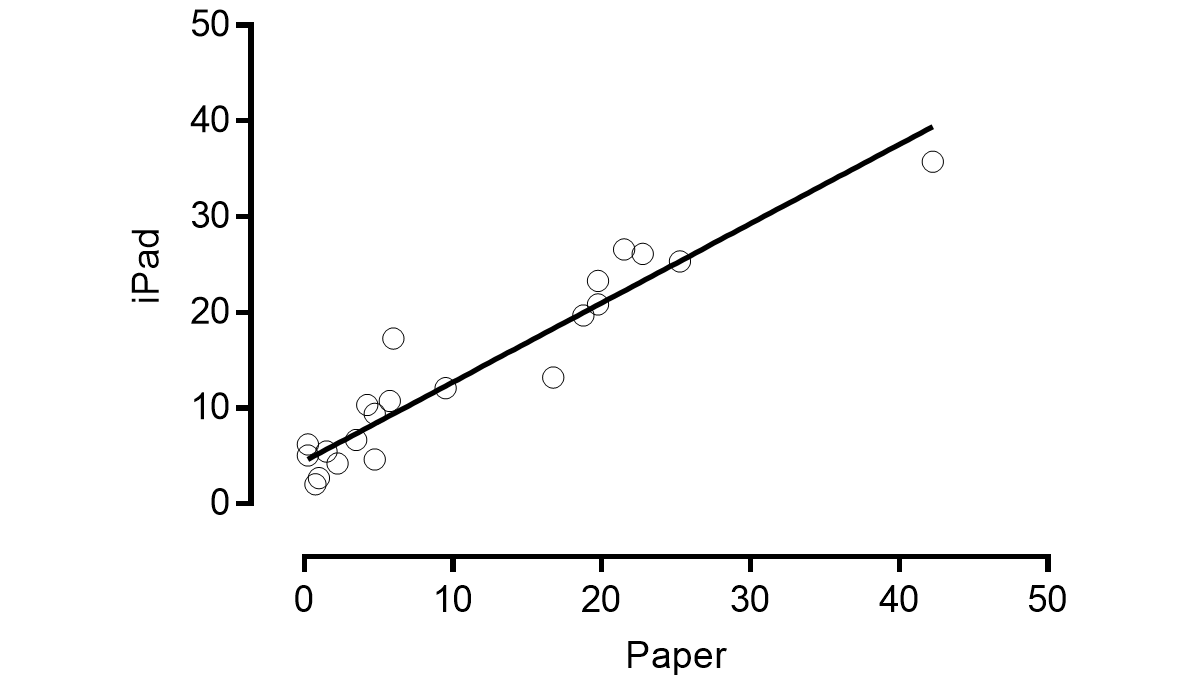
Correlation of scores between paperVAS and iPadVAS measures of pain. Each data point is mean of 4 readings for each participant (data points 22).

A total of 22 community dwelling older adults (4 men, ages 56-86 years) were recruited to test reliability and validity. Grouped (iPad and paper) mean (SD) scores for pain values were 11.9 (10.6). Reliability for both tools was excellent (Table 1). Linear regression demonstrated a strong relationship (*R* ^2^=.904) equation Y=0.8282 × X + 4.451 (Figure 3).

#### Validity

**Figure 4:**
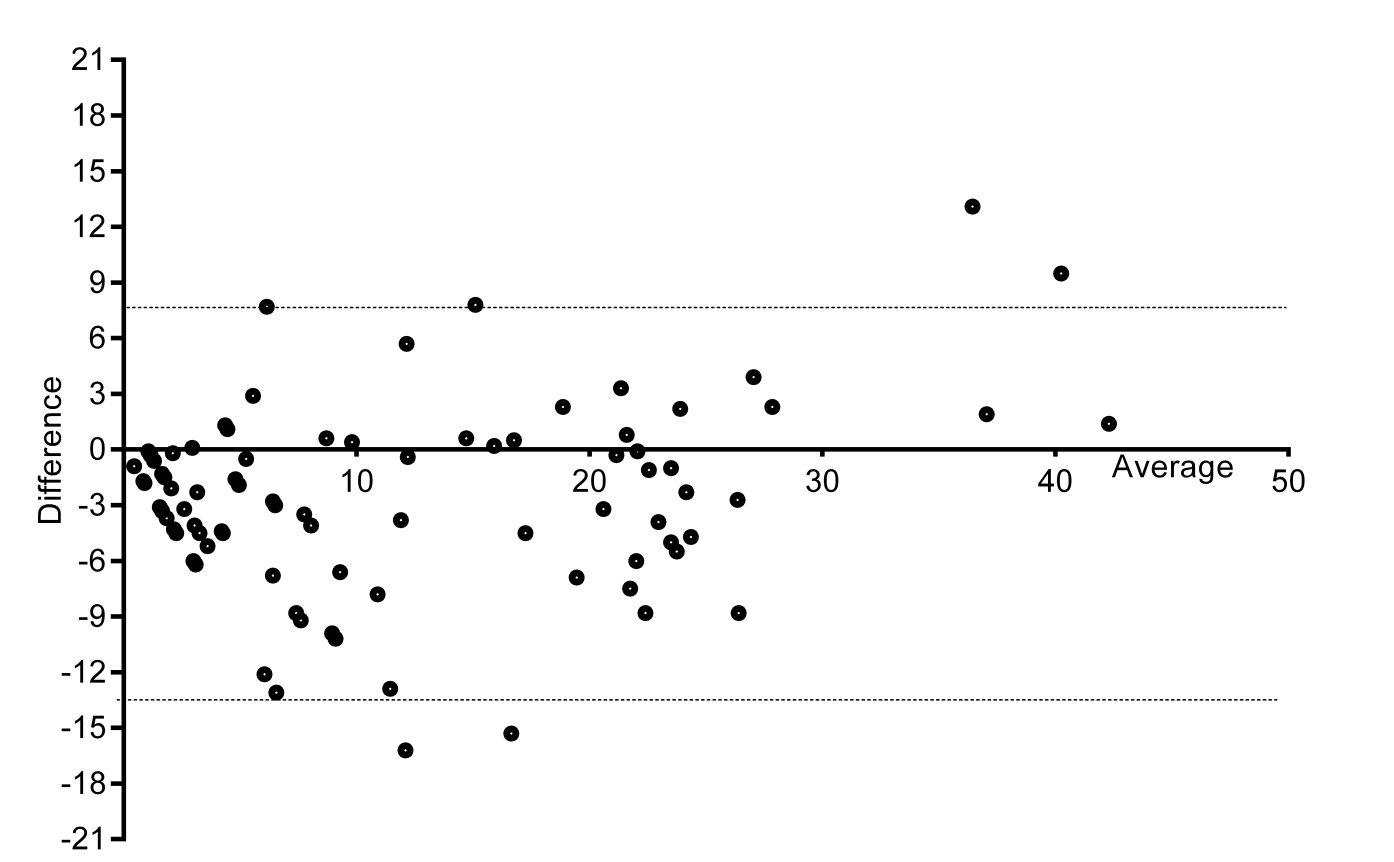
Bland-Altman graph with difference and average of paperVAS and iPadVAS.

There was a small negative bias (SD of bias) toward the iPad (−2.6 [5.0]) with limits of agreement between −12.42 and 7.14 (Figure 4).

**Table 1:** Absolute agreement ICC for the two tools.

Variable (pain)	paperVAS ICC (95%)	iPadVAS ICC (95%)
Individual	0.96 (0.92-0.98)	0.90 (0.82-0.95)
Average	0.99 (0.98-0.99)	0.97 (0.95-1.00)

## Discussion

### Principal Finding

This is the first study to measure accuracy, reliability, and validity of an application on a touch screen iPad for VAS. Accuracy and reliability of the iPadVAS is excellent. Validity shows a small negative bias, but the value of this is not clinically relevant. The iPad application is accurate in that the program reads the same as a manual measurement with a ruler and has a correlation coefficient of .99.

A strength of using the iPad is that it prevents people scoring a line outside the VAS line, which consequently prevents invalid results from being recorded. When using a paper-based version of the VAS, some people indicate their pain levels by drawing less than 0 or more than 100 on the paper. These results can be either interpreted as 0 or 100, respectively, or considered an invalid result. In addition, the thickness of the line drawn by the participant is not affected by finger or stylus width. The high level of similarity of results between paperVAS and iPadVAS indicates that the iPadVAS is a clinically useful tool for collecting both individual and group data.

The magnitude of bias detected in our Bland-Altman analysis is not of clinically relevant amount. A minimum of 13 mm change is required over time to suggest if pain has increased or decreased [6]. This indicates that the small degree of bias (2.6 mm) and the difference in accuracy (with one-third of the data showing 1 mm lower result when measured by the iPad program compared to measured manually) is not of a magnitude to have clinical relevance. These small difference may only be of importance if the VAS is used to determine cut-offs for clients having low-moderate (31-70 mm) or high (more than 70 mm) levels of pain, where 1 mm may make the difference in categorization of pain level.

This new tablet technology is superior to previous electronic data collection tools. The difficulty with personal digital assistants was that the full 100 mm standardized scale could not be used because of the small screen size [8-12]. In some cases, data were collected by a sliding scale or by tapping a number on the screen rather than drawing a line through a line on the screen [8,9]. As well, some studies using these tools did not measure actual perception of pain but rather intensity of different sensations, including cognitive (imagined pain) and sensory stimuli related to heaviness [9] or fatigue [13], impacting the relevance for their use with actual pain perception. Computers, including laptops and Web interfaces, have been used to collect patient data electronically on pain using a VAS [14,15], but more commonly other scales have been used [16-18].

The iPad data collection method has several strengths including portability with large data storage capacity, the ability to simply use Internet access to send data to the health care practitioner, and the potential to interface with other medical records. These features in combination with reduced costs demonstrate that this tool may have the potential to facilitate communication between clinicians and clients while enhancing participatory health care.

For clinicians and researchers, especially those involved in field work, the time, cost, and space savings of data storage are large compared to paper-based copies requiring manual measurement of values and transcription into databases or clinical notes. Hand-held electronic devices collecting questionnaire data show improved documentation completeness and fewer errors than paper-based counterparts [19]. Our study demonstrates similar benefits for VAS, which can now be used confidently for a range of health data collection. This will improve the ability of clinicians to track client health longitudinally, improving individualized clinical decision making. In the future it may be possible to integrate this client data into electronic records, enhancing continuity of care.

Reported benefits for data management [20] and a high patient satisfaction have previously been reported for electronic data collection on computers and laptops; however, the costs associated with that technology was a concern [3]. Newer style tablet devices have reduced costs, improved portability, and enhanced ability of the client to communicate objective data more closely with their healthcare professional.

One benefit of using this technology may be the ability of the devices to provide individuals with a means to objectively monitor and record their pain status without requiring them to attend physical consultation. This is especially important for geographically isolated people and those with limited mobility. The ease of frequent monitoring without the need for recall between consultations may also facilitate regular remote monitoring of chronic health conditions. Similar to other Web-based resources [21,22], this technology gives clients the ability to participate more fully in their health care and may improve the self-efficacy of pain management.

A limitation of our study is that the data were collected in a sample of people from the community who were not in high levels of pain. Replication of this study in participants with moderate to high levels of pain would establish reliability in that population, although previous research indicates that the minimally clinically significant difference in pain scales such as VAS does not differ in populations with different severities of pain [23].

### Conclusion

The iPadVAS provides a convenient, user-friendly, and efficient way of collecting data from participants in measuring their current pain levels. Its use in health care documentation management has the potential to encourage participatory health care. It is accurate, reliable, and valid in healthy older adults.

## Appendix

Multimedia Appendix 1 CONSORT Checklist.

## Abbreviations

ICC: interclass correlation coefficient
VAS: visual analog scales
